# Let-7a inhibits migration, invasion and epithelial-mesenchymal transition by targeting HMGA2 in nasopharyngeal carcinoma

**DOI:** 10.1186/s12967-015-0462-8

**Published:** 2015-03-31

**Authors:** Aibing Wu, Kunpeng Wu, Jinmei Li, Yanli Mo, Yanming Lin, Yuzhou Wang, Xiang Shen, Shujun Li, Lixia Li, Zhixiong Yang

**Affiliations:** Oncology Center, Affiliated Hospital of Guangdong Medical College, No.57 Peoples Avenue South, Zhanjiang, Guangdong 524002 People’s Republic of China

**Keywords:** MicroRNA, Let-7a, HMGA2, Nasopharyngeal carcinoma, Metastasis

## Abstract

**Background:**

Let-7a has been shown to play important roles in nasopharyngeal carcinoma (NPC) cell proliferation and apoptosis, but little is known about the function and mechanism of let-7a in nasopharyngeal carcinoma metastasis. We aimed to investigate the function and mechanism of let-7a in nasopharyngeal carcinoma metastasis and clarified the regulation of high mobility group A2 (HMGA2) by let-7a.

**Methods:**

The expression levels of let-7a and HMGA2 were examined in NPC clinical specimens using quantitative reverse transcription-PCR (RT-qPCR). HMGA2 was confirmed as a target of let-7a through luciferase reporter assays, RT-qPCR, and Western blotting. Furthermore, the roles of let-7a and HMGA2 in regulating NPC cells biological properties including proliferation, migration, invasion and epithelial-mesenchymal transition (EMT) process were analyzed with let-7a mimics and si-HMGA2 transfected cells.

**Results:**

Our study demonstrated that let-7a was downregulated and inversely associated with the clinical stage, T classification and N classification, and HMGA2 was upregulated and directly associated with the clinical stage and N classification in patients with NPC. Moreover, there was an inverse correlation between let-7a expression and HMGA2 expression in NPC patient. In addition, HMGA2 was negatively regulated at the posttranscriptional level by let-7a via a binding site of HMGA2-3′UTR. In addition, synthetic let-7a mimics suppressed NPC cells migration, invasion and EMT process and knockdown of HMGA2 was consistent with the effects of let-7a in NPC cells.

**Conclusion:**

Let-7a directly downregulates HMGA2 protein expression, which suppress NPC cell migration, invasion and EMT process. Let-7a could serve as a potential diagnostic marker and therapeutic target for NPC.

**Electronic supplementary material:**

The online version of this article (doi:10.1186/s12967-015-0462-8) contains supplementary material, which is available to authorized users.

## Background

Nasopharyngeal carcinoma (NPC) is a malignant disease with a distinct geographical and ethnic distribution. The global statistics of cancer showed its distinctly unbalanced endemic distribution, with the highest incidence in Southern China [1]. Unfortunately, the majority of NPC patients tend to present a more advanced stage of disease when first diagnosed because of its vague symptoms and deep location. Although NPC patients are sensitive to radiotherapy, treatment failure remains high due to the development of local recurrence and distant metastasis [2]. Because the mechanisms of NPC metastasis that are not completely understood to date, further investigation of this mechanism is imminently needed.

MicroRNAs (miRNAs) are endogenous non-coding small RNAs about 19–25 nucleotide long, which contribute to the regulation of their target genes mRNA by usually base-pairing to the 3′-untranslated region (3′-UTR), and results in either mRNA degradation or translation inhibition [3-5]. It has been reported that miRNAs can control a variety of biological processes including cellular differentiation, proliferation, cell mobility and cell death [6,7]. Moreover, recent evidence indicated that miRNAs can function either as tumor suppressors or oncogenes in tumor progression [8,9]. Recent studies reported that several miRNAs are related to NPC development and progression by regulating cell growth, metastasis, and apoptosis [10-13], indicating that miRNAs play significant roles in NPC tumorigenesis. However, no study has elucidated the functions and mechanisms of let-7 miRNA family in NPC metastasis.

Let-7 was first identified in Caenorhabditis elegans and is highly conserved in C.elegans, Drosophila, Zebrafish, and Humans [3]. Let-7 scarcely expresses in embryonic stages, but obviously enhances in mature and differentiated tissues [14]. Moreover, let-7 has been shown as being decreased during cancer progression in various human cancers [3]. Recent studies have indicated that let-7 regulated expression of certain oncogenes and contributed to carcinogenesis, in lung [15], colon [16], head and neck [17], and pancreatic cancer [18]. In addition, several studies further revealed that let-7 plays important roles in the regulation of metastasis and epithelial-mesenchymal transition (EMT) in breast cancer [19], oral cancer [20] and esophageal cancer [21]. Interestingly, downregulation of let-7 initiated and maintained oncostatin M-induced EMT phenotypes in breast cancer, and that high mobility group A2 (HMGA2) acted as a switching actor in this progress [19].

HMGA2 is a small nonhistone chromosomal protein that has no intrinsic transcriptional activity, but can modulate transcription by altering chromatin architecture [22]. HMGA2 proteins are involved in many diverse biological processes such as differentiation, embryogenesis, and neoplastic transformation [23]. In contrast with let-7, HMGA2 was overexpressed in embryonic and carcinoma tissue and was associated with highly malignant phenotype and was a poor prognostic factor [24]. Three miRNAs target databases (TargetScan, miRanda and PicTar) predicted HMGA2 to be a potential target of let-7. Although the direct regulatory relationship between let-7a and HMGA2 has been confirmed in lung cancer [25], breast cancer [19], and esophageal cancer [21], little is known about let-7 and HMGA2 in NPC.

In this study, we aimed to investigate the role of let-7a and HMGA2 in NPC. We found that downregulation of let-7a and upregulation of HMGA2 in NPC tissues compared with normal tissues, and associated with clinical stage and N classification in NPC. Moreover, there was an inverse correlation between let-7a expression and HMGA2 expression in NPC patient. In addition, synthetic let-7a mimics inhibited the migration, invasion, and EMT of NPC cells in vitro. Furthermore, we validated HMGA2 was a target of let-7a by 3′-UTR luciferase assays and western blot analysis. Meanwhile, knockdown of HMGA2 was consistent with the effects of let-7a in NPC cells, but no influence on the expression of let-7a. Our study demonstrated that let-7a targets HMGA2 to regulate migration and invasion through epithelial-mesenchymal transition in NPC.

## Materials and methods

### Clinical specimens

A total of 48 freshly-frozen NPC samples and 20 normal nasopharyngeal epithelium samples were collected from the Affiliated Hospital of Guangdong Medical School between March 2013 and March 2014. No patients had received any form of tumour-specific therapy before diagnosis. Before the use of these clinical samples, prior consents from the patients and approval from the Institutional Ethics Committee of the Affiliated Hospital of Guangdong Medical School were obtained (HM-81201672). The histopathological diagnosis of all samples was respectively diagnosed by two pathologists. The clinical staging was based on the 7th edition of the AJCC Cancer Staging Manual. The main demographic and clinicopathological characteristics were presented in Table 1.

**Table 1:** **Correlation between the clinicopathologic characteristics and expression of let-7a and HMGA2 in nasopharyngeal carcinoma**

**Characteristics**	**n**	**Let-7a**		***p***	**HMGA2**		***p***
		**High, n(%)**	**Low, n(%)**		**High, n(%)**	**Low, n(%)**	
Gender							
Male	28	14(50.0)	14(50.0)	1.000	12(42.9)	16(57.1)	0.063
Female	20	10(50.0)	10(50.0)		14(70.0)	6(30.0)	
Age(y)							
≥50	24	11(45.8)	13(54.2)	0.564	10(41.7)	14(58.3)	0.082
<50	24	13(54.2)	11(45.8)		16(66.7)	8(33.3)	
Clinical stage							
I-II	16	12(75.0)	4(25.0)	0.014	5(31.2)	11(68.8)	0.024
III-IV	32	12(37.5)	20(62.5)		21(65.6)	11(34.4)	
T classification							
T1-T2	21	15(71.4)	6(28.6)	0.009	9(42.9)	12(57.1)	0.165
T3-T4	27	9(33.3)	18(66.7)		17(63.0)	10(37.0)	
N classification							
N0-N1	19	13(68.4)	6(31.6)	0.039	5(26.3)	14(73.7)	0.002
N2-N3	29	11(37.9)	18(62.1)		21(72.4)	8(27.6)	
Distant metastasis							
No	44	24(54.5)	20(45.5)	0.117	23(52.3)	21(47.7)	0.727
Yes	4	0(0)	4(100)		3(75.0)	1(25.0)	

### Cell culture

All other cell lines were from the Cell Bank of Type Culture Collection of the Chinese Academy of Sciences (Shanghai, China). Five NPC cell lines HONE1, CNE1, CNE2, 5-8F and 6-10B were maintained in RPMI 1640 medium supplemented with 10% fetal bovine serum (FBS; Biological Industries, Israel). All the cell lines were incubated in a humidified chamber with 5% CO_2_ at 37°C, and used for this study within 6 months of resuscitation.

### Real-time quantitative reverse transcription-polymerase chain reaction (RT-qPCR)

To quantitate mRNA expression, total RNA was extracted from clinical samples and NPC cell lines with RNAiso Plus (Takara, Japan). The isolated total RNA was reverse transcribed using the One Step PrimeScript miRNA cDNA Synthesis Kit (Takara, Japan) for Let-7a and the PrimeScript RT Master Mix (Perfect Real Time) (Takara, Japan) for HMGA2, according to manufacturer instructions. Relative expression was calculated via the comparative cycle threshold (Ct) method and was normalized to the expression of U6 small RNA or β-actin. The sequence-specific forward primers for mature Let-7a and U6 internal control were 5′-GGTGAGGTAGTAGGTTGTATAGTT-3′ and 5′-CTCGCTTCGGCAGCACATATA-3′, respectively. The Uni-miR qPCR Primer was included in the kit. Forward and reverse primers sequences for HMGA2 mRNA were 5′- CTCAAAAGAAAGCAGAAGCCACTG -3′ and 5′- TGAGCAGGCTTCTTCTGAACAACT −3 respectively. Forward and reverse primers sequences for β-actin mRNA were 5′-GGCGGCAACACCATGTACCCT-3′ and 5′-AGGGGCCGGACTCGTCATACT-3 respectively. qPCR was performed using SYBR Premix Ex Taq™ II (Takara, Japan) on a LightCycler (Roche Diagnostics, USA). Relative quantification of miRNA expression was calculated by using the 2^-△△Ct^ method. The raw data were presented as the relative quantity of target miRNA or mRNA, normalized with respect to U6 or β-actin, and relative to a calibrator sample. All qRT-PCR reactions were performed in triplicate.

### miRNAs, small interfering RNAs and transfection

Let-7a mimic and the negative control were obtained from RiboBio (Guangzhou, China). Small interfering RNAs (siRNAs), HMGA2-siRNA 1, HMGA2-siRNA 2, HMGA2-siRNA 3 and the negative control siRNA were also obtained from RiboBio (Guangzhou, China). For convenience, Let-7a mimic negative control and negative control siRNA are termed Let-7a mimics-NC and NC-siRNA. miRNAs and siRNAs transfection was performed using riboFECT™ CP (RiboBio, China) according to the manufacturer’s instruction. The relative level of Let-7a and HMGA2 in transfected cells were examined by qRT-PCR. (Total RNAs and protein were prepared 48 h after transfection for RT–qPCR and western blotting analysis.)

### Luciferase reporter assays

The HMAGA2 wild-type (Wt) and mutant (Mt) 3′UTR were created and cloned to the firefly luciferase-expressing vector pLUC (Additional file 1: Figure S2). For the luciferase assay, CNE-2 and 5-8F cells were seeded triplicate in 12-well plates the day before transfection, and co-transfected with the HMAGA2 Wt or Mt 3′UTR reporter vector, and Let-7a mimics or Let-7a mimics-NC using riboFECT™ CP (RiboBio, China). After 48 hours of transfection, the cells were harvested and lysed, and the luciferase activities were assayed using the Dual-Luciferase Reporter System (Promega, USA). Three independent experiments were performed.

### Cell proliferation assay

Cell proliferation was analyzed using MTT assay. Briefly, 1 × 10^3^ cells were seeded into a 96-well plate with quadruplicate repeat for each condition. For si-MMP14 and si-control, the cells were incubated for 1, 2, 3, and 4 days. Twenty microliters of MTT (5 mg/ml) (Sigma, USA) was added to each well and incubated for 4 h. At the end of incubation, the supernatants were removed and 150 μl of DMSO (MP, USA) was added to each well. The absorbance value (OD) of each well was measured at 490 nm. Experiments were performed three times.

### Cell migration and invasion assays

After 48 h transfection, cells were resuspended into serum-free medium. For migration assays, 5.0 × 10^4^ cells were placed in the top chamber of each insert (Corning Costar, USA) with 8.0 μm pores; for invasion assays, 1.0 × 10^5^ cells were seeded in a Matrigel-coated chamber (Corning Costar, USA). In the lower chamber, 600 μl of RPMI 1640 with 10% FBS was added as a chemoattractant. After the cells were incubated for 24 h, the insert was washed with PBS, and cells on the upper surface of the membrane were removed with a cotton swab. Cells adhering to the lower surface were fixed with methanol, stained with Giemsa. The number of cells in the membrane was counted from 5 randomly selected visual fields with a microscope at 100× magnification. All assays were independently repeated at least three times.

### Western blotting

Transfected CNE-2 and 5-8F cells were cultured for 72 hours and then harvested on ice using RIPA lysis buffer (Cwbiotech, China). Total protein concentrations were measured using the BCA protein assay kit (Cwbiotech, China). Total protein was separated by SDS-PAGE using 8-12% polyacrylamide gel and transferred to polyvinylidenefluoride membrane (PVDF; Millipore, USA). The membrane was immunoblotted overnight at 4°C with primary antibodies: anti-HMGA2, anti-E-Cadherin, anti-ZO-1, anti-MMP-2, anti-MMP-9, anti-Vimentin, anti-Snail, Slug, anti-β-Catenin(1:1000 dilution; Cell Signaling Technology, USA), and anti-β-actin (1:2000 dilution; Cwbiotech, China). The secondary antibody, horseradish peroxidase-conjugated goat IgG (1:1000 dilution; Cell Signaling Technology, USA), was incubated with the membrane for 1 h after 3 washes with TBST. Signals were detected with ECL detection reagent (Cwbiotech, China). The images were obtained and quantified by Quantity One (Bio-Rad, USA). Each experiment was performed in triplicates.

### Statistical analysis

Data were presented as mean ± SD. The Student’s *t* test was used for comparisons of two independent groups. One-way ANOVA was used to determine cell growth in vitro. The Chi-square test was applied to the examination of relationship between let-7a and HMGA2 expression and clinicopathologic characteristics. All statistical analysis was performed with SPSS 17.0 software, and *P* values of < 0.05 were defined as statistically significant.

## Results

### Let-7a was downregulated and HMGA2 was upregulated in NPC clinical specimens

**Figure 1:**
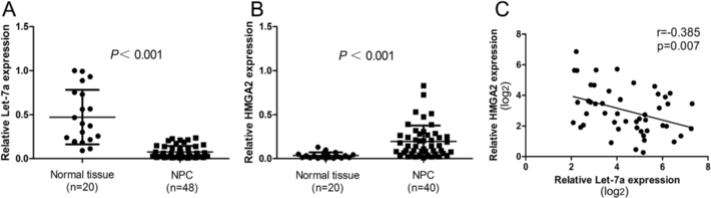
**Expression of let-7a and HMGA2 in NPC clinical samples. (A-B)** Let-7a was downregulated and HMGA2 was upregulated in NPC (n = 48) compared with normal nasopharyngeal epithelial tissues (n = 20). Data is presented as the mean ± SD, and P values were calculated with the Student *t*-test. **(C)** Significant correlations between the expression of let-7a and HMGA2 in NPC (n = 48) were demonstrated using the Pearson’s correlation coefficient analysis (r = −0.385, *P* = 0.007).

In this study, we firstly tested let-7a expression in 48 freshly-frozen NPC and 20 normal nasopharyngeal tissue samples. Compared with normal nasopharyngeal epithelial tissues, NPC tissues showed lower expression levels of let-7a and higher expression levels of HMGA2 mRNA. (Figure 1A-B, both *P* < 0.001).

### Relationship between clinicopathological characteristics and the expression of let-7a and HMGA2 in NPC patients

In this study, patients with values less than the average expression level of let-7a and HMGA2 were assigned to a low expression group, whereas those with expression values above average were assigned to a high expression group. The cut-off levels were 4.41 for let-7a (normalized to U6), and 3.01 for HMGA2 (normalized to β-actin), which is the mean level of relative quantity. This classification was based on published studies [26,27]. The relationships between clinicopathological characteristics and let-7a and HMGA2 expression levels in individuals with NPC are summarized in Table 1. We observed that the expression level of let-7a was positively correlated with the status of clinical stage (I-II vs. III-IV, *P* = 0.014), T classification (T1-T2 vs. T3-T4, *P* = 0.009), and N classification (N0-N1 vs. N2-N3, *P* = 0.039) in NPC patients. However, we did not find a significant association of let-7a expression levels with patient’s gender (Male vs. Female, *P* = 1.000), age (≥50 vs. <50, *P* = 0.564), and distant metastasis (Yes vs. No, *P* = 0.117). In addition, there were significant correlations between HMGA2 expression and clinical staging (I-II vs. III-IV, *P* = 0.024), and N classification (N0-N1 vs. N2-N3, *P* = 0.002) in NPC patients. However, HMGA2 expression was not associated significantly with gender (Male vs. Female, *P* = 0.063), age (≥50 vs. <50, *P* = 0.082), T classification (T1-T2 vs. T3-T4, *P* = 0.165), distant metastasis (Yes vs. No, *P* = 0.727).

### Inverse correlation between let-7a expression and HMGA2 expression in NPC patients

In 48 NPC patients, the inverse correlation between the expression of let-7 and HMGA2 in NPC was confirmed using Pearson’s correlation coefficient analysis (r = −0.385, *P* = 0.007, Figure 1C) and Spearman’s correlation coefficient analysis (*P* = 0.012).

### Expression of let-7a in NPC cell lines

**Figure 2:**
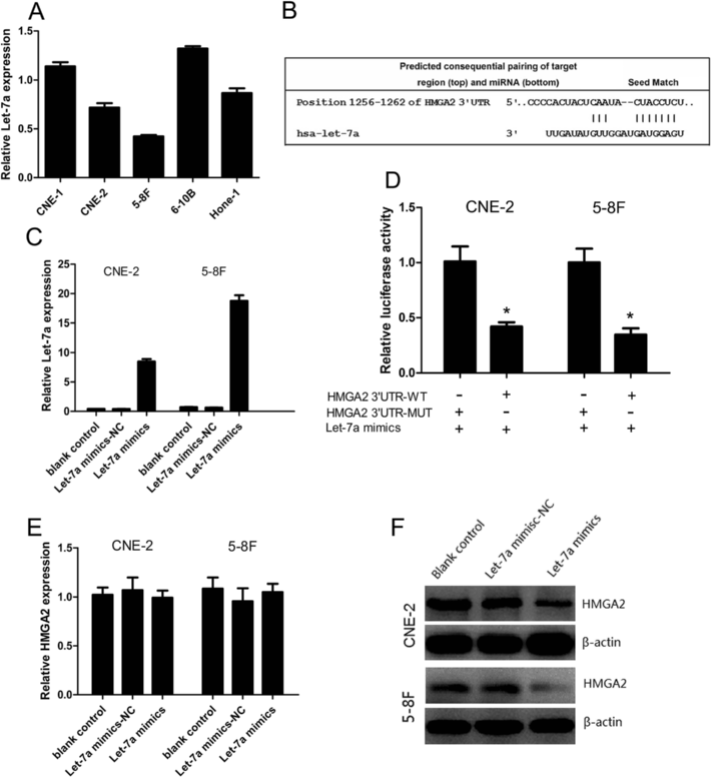
**Let-7a inhibited the protein expression of HMGA2 by binding to its 3′UTR. (A)** RT-qPCR of let-7a expression in NPC cell lines with different degrees of differentiation and metastasis ability. **(B)** CNE-2 and 5-8F cells transfected with the let-7a mimics showed significantly increased let-7a expression compared to controls. **(C)** Position of the let-7a target site in 3′UTR of HMGA2 mRNA predicted by TargetScan. **(D)** Relative luciferase activity of CNE-2 and 5-8F cells after co-transfection with wild type (WT) or mutant (MUT) HMGA2 3′UTR reporter genes and let-7a mimics or control. **(E-F)** Protein and mRNA expression levels of HMGA2 are tested after let-7a transfected in CNE-2 and 5-8F by western blotting and RT-qPCR. *, *P* < 0.001 compared with control.

We first analyzed the expression level of let-7a in a panel of NPC cell lines with different degrees of differentiation and metastatic ability including CNE-1 (high differentiation), CNE-2(low differentiation), 5-8F (high metastatic ability), 6-10B (low metastatic ability), HONE-1(low differentiation). We observed that let-7a expression was relatively lower in CNE-2 cells than in CNE-1 and HONE-1 cells, and also was lower in 5-8F cells than in 6-10B cells (Figure 2A), suggesting that let-7a expression may be associated with the degree of NPC cell differentiation and metastatic ability. Based on this expression pattern, we therefore chose CNE-2 and 5-8F cells for the following gain-of-function studies.

### Let-7a inhibited the protein expression of HMGA2 via binding to its 3′UTR

Based on the miRanda and TargetScan software, potential binding sites of let-7a in the 3′UTR of HMGA2 were predicted (Figure 2B). The successful overexpressions of let-7a in the CNE-2 and 5-8F cells were confirmed by RT-qPCR (Figure 2C). We then performed a luciferase reporter assay to prove that let-7a directly targets HMGA2. We found that co-transfection of let-7a mimics and pLUC-HMGA2-wt significantly decreased the luciferase activity in CNE-2 and 5-8F cells as compared with the control. Moreover, let-7a mimics had no effect on the luciferase activity when co-transfected with pLUC-HMGA2-mut (Figure 2D). These data showed that HMGA2 is one of direct targets of let-7a. We further analyzed the HMGA2 mRNA and protein expression by using RT-qPCR and western blotting respectively after transfecting CNE-2 and 5-8F cells with let-7a mimics. The increase let-7a levels significantly decreased HMGA2 protein expression at CNE-2 and 5-8F as determined by western blotting (Figure 2F, Additional file 2: Figure S1A), while mRNA remained unchanged (both *P* > 0.05) (Figure 2E).

### HMGA2 is regulated uniaxially by let-7a, but not feedback regulatory loop

**Figure 3:**
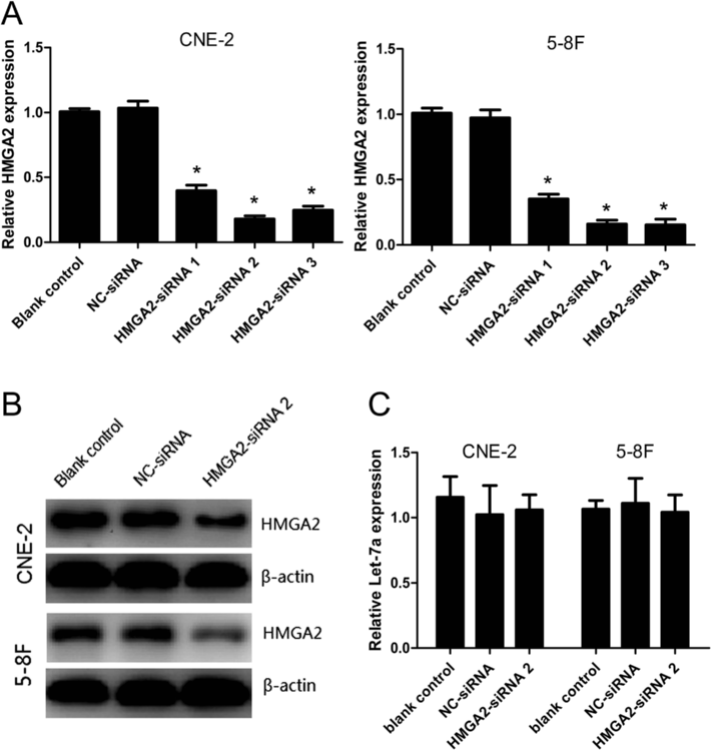
**HMGA2 is regulated uniaxially by Let-7a but not feedback regulatory loop. (A)** The efficiency of HMGA2-siRNA was distinguished by RT-qPCR in NPC cells and HMGA2 si-RNA 2 was most effective both CNE-2 and 5-8F cells. **(B)** Analysis of protein expression by western blot, HMGA2 protein is reduced by small interfering RNA (HMGA2-siRNA 2) in CNE-2 and 5-8F cells. **(C)** Knocking down the expression of HMGA2 had no alternation on let-7a level. *, P < 0.001 compared with control.

The efficiency of HMGA2-siRNA was distinguished by RT-qPCR and confirmed by western blotting. As shown in Figure 3A-B, the relative expression of HMGA2 mRNA is the lower in the group of HMGA2-siRNA 2 after CNE-2 and 5-8F cells were transfected with si-HMGA2 for 48 h, and HMGA2 protein expression of was significantly lower in CNE-2 and 5-8F cells transfected with the HMGA2-siRNA 2 than with NC-siRNA or blank control. Interestingly, when we treated CNE-2 and 5-8F cells with a let-7a mimic, we found that the HMGA2 protein expression was significantly decreased, supposing whether HMGA2 was also able to regulate the expression of let-7a via a feedback loop in NPC cells. However, we found that knock-down HMGA2 had no effect on the let-7a expression at CNE-2 and 5-8F cells (Figure 3C). Thus, there is no feedback regulatory loop between let-7a and HMGA2 in NPC cells.

### Let-7a suppressed NPC cells proliferation, migration and invasion

**Figure 4:**
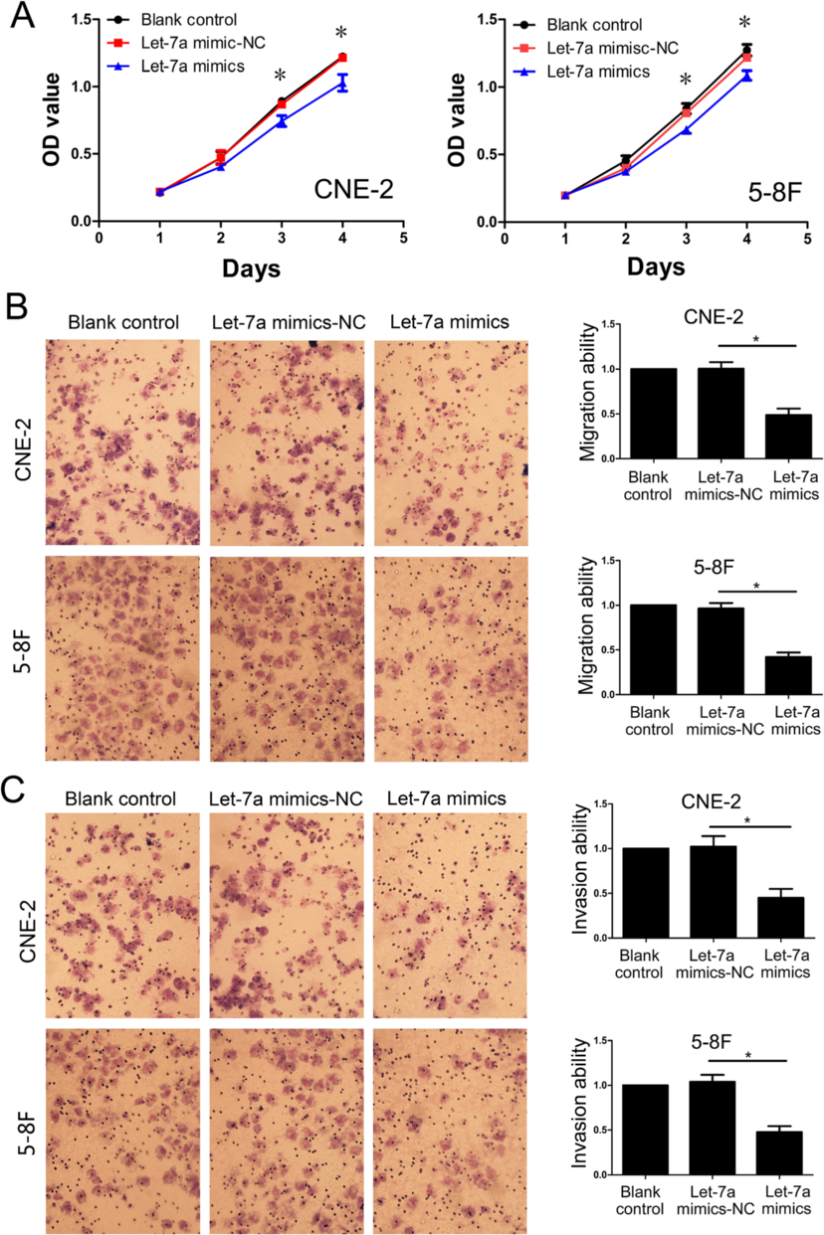
**Overexpression of let-7a suppressed NPC cells proliferation, migration and invasion. (A)** The growth curves determined by MTT assay showed that let-7a mimics inhibited NPC cells growth compared with negative control of let-7a. **(B)** Up-regulated let-7a expression dramatically decreased the ability of CNE-2 and 5-8F cells migration in vitro. **(C)** Elevated let-7a expression inhibited invasiveness of CNE-2 and 5-8F cells. The results were expressed as fold change relative to the corresponding blank control. Data is presented as the mean ± SD. *, *P* < 0.001 compared with control.

We examined the effect of increased let-7a expression on NPC cell growth in vitro. The growth curves determined by MTT assay showed that let-7a inhibited NPC cell growth compared with negative control of let-7a (Figure 4A). To evaluate the impact of let-7a on cell migration and invasion, the cell migration and invasion assay were employed. In transwell migration assay, we found that overexpression of let-7a reduced CNE-2 and 5-8F cells migration (Figure 4B, *P* < 0.001). Consistent with this finding, transwell invasion assay showed that let-7a mimics significantly inhibited invasion capacity of CNE-2 and 5-8F cells (Figure 4C, *P* < 0.001).

### HMGA2 is involved in the regulation of cell migration and invasion by let-7a

**Figure 5:**
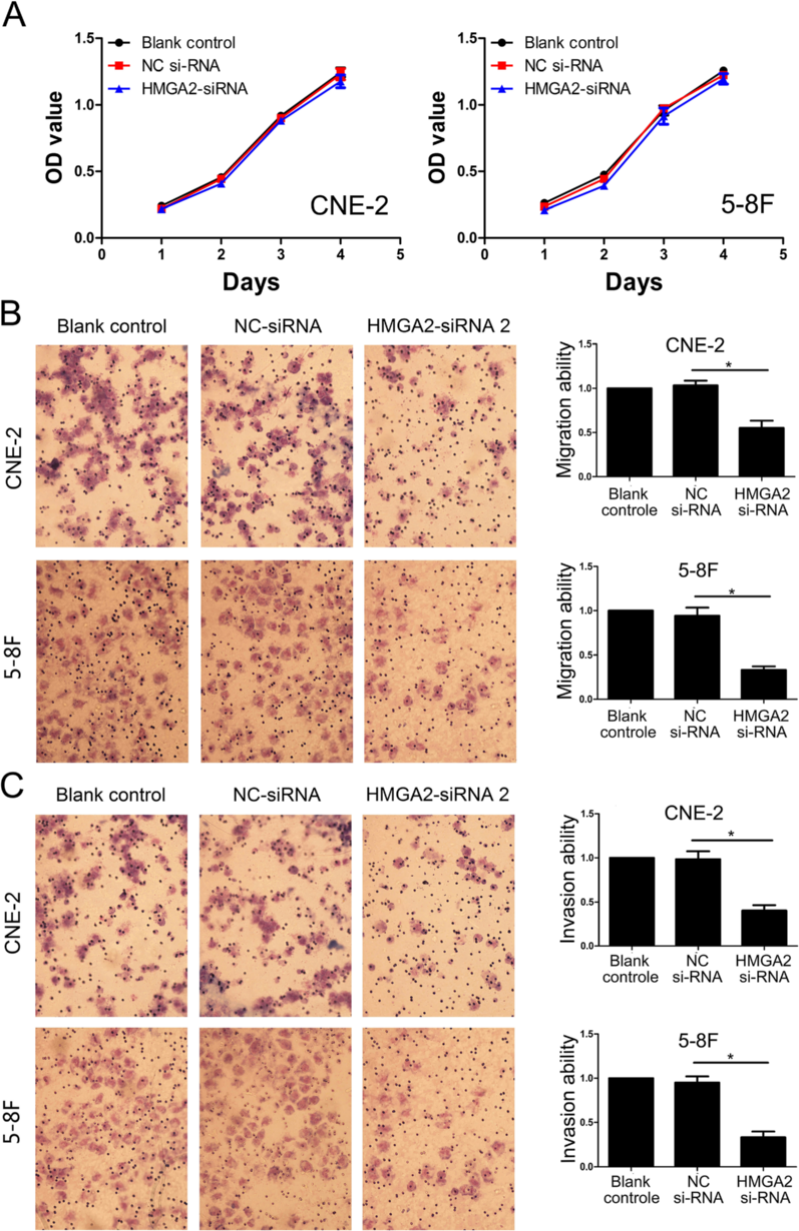
**Knockdown of HMGA2 suppressed NPC cells migration and invasion. (A)** The growth curves determined by MTT assay showed that knockdown of HMGA2 had no effect on NPC cells growth. **(B)** Down-regulated HMGA2 expression dramatically decreased the ability of CNE-2 and 5-8F cells migration in vitro. **(C)** Suppressed HMGA2 expression inhibited invasiveness of CNE-2 and 5-8F cells. The results were expressed as fold change relative to the corresponding blank control. Data is presented as the mean ± SD. *, *P* < 0.001 compared with control.

**Figure 6:**
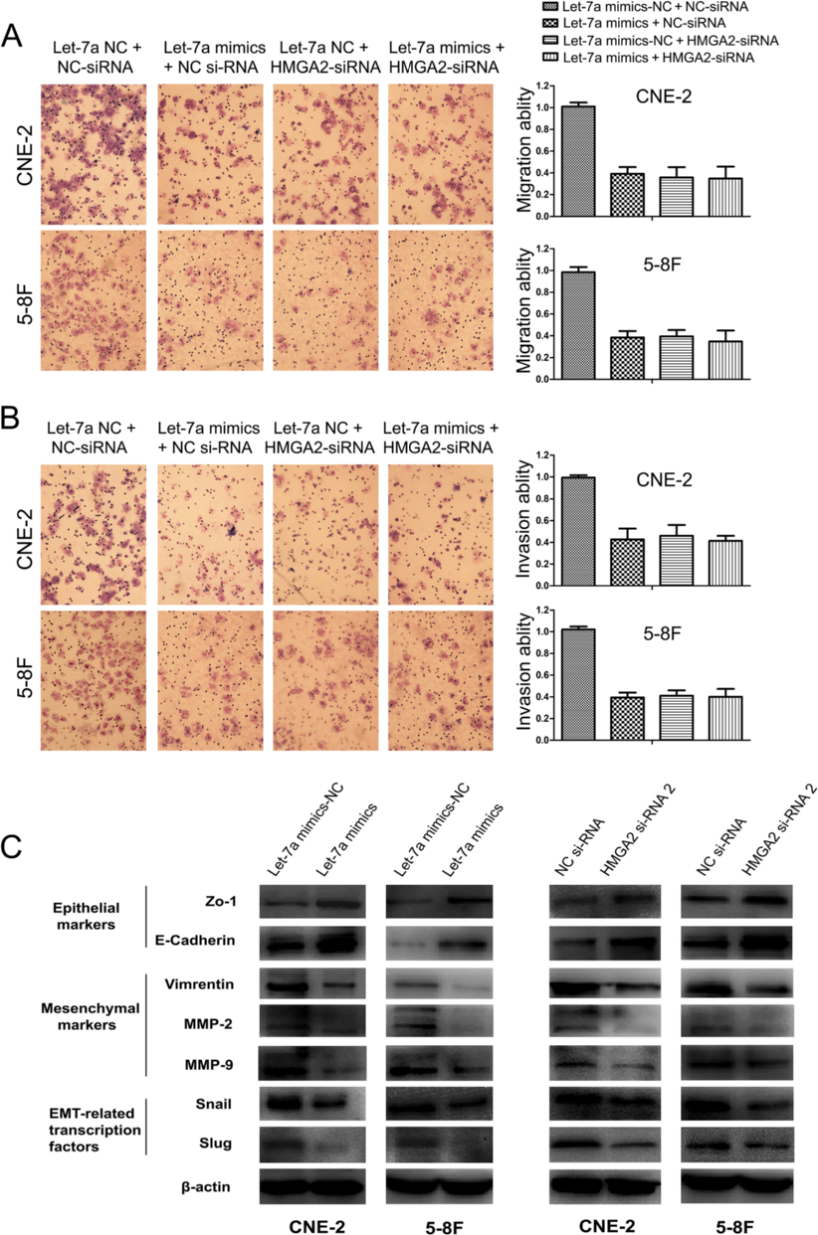
**HMGA2 is a functional target of let-7a to regulate NPC cells migration and invasion and expression of EMT-associated genes. (A)** The migration assay suggested that co-transfection of let-7a mimics and HMGA2-siRNA didn’t profoundly reduce NPC cells migration in comparison to HMGA2-siRNA+let-7a mimics-NC and NC-siRNA+let-7a mimics. **(B)** The invasion assay indicated that co-transfection of let-7a mimics and HMGA2-siRNA didn’t further suppress thfce invasiveness of NPC cells compared with HMGA2-siRNA+let-7a mimics-NC and NC-siRNA+let-7a mimics. **(C)** Transfection of let-7a mimics reduced the expression of EMT-marker genes including Vimentin, MMP2, MMP9, Snail and Slug, and promoted E-cadherin and Zo-1 expression. Knocking down endogenous HMGA2 expression decreased the expression of EMT-marker genes including Vimentin, MMP2, MMP9, Snail and Slug, and increased E-cadherin and Zo-1 expression.

Because let-7a inhibits NPC cell proliferation, migration and invasion and suppresses HMGA2 protein expression, we are interested in exploring whether let-7a functions in cell proliferation, migration and invasion via targeting to HMGA2. To examine the role of HMGA2 in NPC cell migration and invasion, a siRNA against HMGA2 was introduced into CNE-2 and 5-8F cells to reduce HMGA2 expression (Figure 3B, Additional file 2: Figure S1B). HMGA2 silencing has no effect on NPC cell growth (Figure 5A), but significantly decreased the migration and invasion of CNE-2 and 5-F8 cells (Figure 5B-C), which was similar to the phenotype of migration and invasion induced by let-7a (Figure 4B-C). To further verify whether let-7a regulated cell migration and invasion through HMGA2, we co-transfected let-7a mimics and HMGA2-siRNA into CNE-2 and 5-8F cells. We found that co-transfection of let-7a mimics and HMGA2-siRNA didn’t profoundly reduced cell migration and invasion in NPC cells (Figure 6A-B). These findings suggested that HMGA2 is a functional mediator for let-7a in NPC cells.

### Let-7a and HMGA2 regulated epithelial-mesenchymal transition in NPC cells

To further study the mechanism by which let-7a and HMGA2 regulate cell migration and invasion, we examined protein levels of EMT-associated genes in NPC CNE-2 and 5-8F cells with suppressed let-7a and HMGA2 expression respectively. We found that let-7a mimics decreased the expression of MMP2, MMP9 and EMT-marker genes including Snail, Slung, and Vimentin and increased E-cadherin and Zo-1 expression (Figure 6C, Additional file 2: Figure S1C). Similarly, knocking down endogenous HMGA2 expression suppressed the activation of MMP2, MMP9 and EMT-marker genes including Snail, Slug, and Vimentin and increased E-cadherin and Zo-1 expression, and elevated the expression of E-cadherin and Zo-1 (Figure 6C, Additional file 2: Figure S1C).

## Discussion

Recognition of cancer-specific miRNAs and their targets is critical for understanding their roles in tumor development and progression, and may be significant for exploring novel therapeutic targets. Several reports indicated that miRNAs were abnormally expressed in NPC [28,29], and the dysregulated miRNAs could regulate NPC cell growth and metastasis [10-13]. Let-7a was reported to be frequently downregulated in several types of cancers, such as lung cancer [15], colon cancer [16], head and neck cancer [17], and pancreatic cancer [18]. Moreover, there were two studies shown the dysregulation of let-7a expression was involved in NPC cell proliferation and apoptosis [30,31], but little is known about the function and mechanism of let-7a involving in NPC metastasis.

Similar to a report from Cai et al. [31], we found that let-7a was downregulated in NPC clinical samples and further presented the evidence that let-7a expression was positively correlated with the status of clinical stage, T classification, and N classification in NPC patients. Meanwhile, we firstly found HMGA2, which predicted as the target of let-7a, was overexpression in NPC tissues compared with normal nasopharyngeal epithelial tissues and correlated with the status of clinical stage and N classification, but no T classification. Moreover, there was an inverse correlation between let-7a expression and HMGA2 expression in NPC patient. Similarly, the significant inverse association was also detected between let-7 and HMGA2 in esophageal cancer [21], retinoblastomas [32] and pituitary adenomas [33]. Therefore, we supposed that let-7a regulated NPC cell metastasis through targeting HMGA2.

In our study, synthetic let-7a mimics inhibited NPC cells migration and invasion and knockdown of HMGA2 was consistent with the effects of let-7a in NPC cells. Moreover, HMGA2 was identified as a direct and functional target of let-7a via binding to the 3′-UTR of HMGA2. In lung cancer, Wang et al. reported that let-7a inhibited the proliferation and invasion of lung cancer cell line by inhibiting HMGA2 and K-RAS protein expression [34]. Recent studies have shown several members of let-7 family were diminished expression in breast cancer compared with normal breast tissues, and inhibited the breast cancer cell migration and invasive ability through regulating HMGA2, Lin28, GAB2, FN1, MAPK and MMPs [19,35,36]. Furthermore, we observed significantly diminished expression of let-7a only in protein level, but no mRNA level. Our results of the inconsistencies between the mRNA and protein levels of HMGA2 indicated that let-7a regulates HMGA2 expression at a posttranscriptional level, which is consistent with the mechanism of miRNAs [37]. Lin28 is also a target of let-7a and mainly plays an important role in cell stemness [38]. Interestingly, the double-negative feedback loop between let-7 and Lin28 was found in tumor cells [38]. Whether there is a double-negative feedback loop between let-7 and HMGA2 in NPC cells was an interesting problem. However, our results revealed knockdown of HMGA2 has no effect on the let-7a expression. Thus, there was just a unidirectional regulation between let-7a and HMGA2 in NPC cells.

Epithelial-mesenchymal transition (EMT) is a critical process by which epithelial cells lose their epithelial morphology and acquire a mesenchymal phenotype, characterized by the decrease of epithelial proteins such as E-cadherin and Zo-1, and the increase of mesenchymal proteins such as vimentin and fibronectin [39,40]. Snail and Slug are members of zinc finger family and play a central transcriptional role in the regulation of EMT by binding directly to specific E-boxes on the E-cadherin promoter [41,42]. It is widely accepted that EMT plays a significant role during tumor invasion and metastasis, and aggressive cancer cells often present with a loss of epithelial characteristics and acquire a mesenchymal phenotype [43]. Chang et al. reported that overexpression of let-7 effectively reversed the EMT phenotype, blocked migratory and invasive abilities in oral cancer cells [20]. Li et al. found that 3, 3′-diindolylmethane and isoflavone can cause up-regulation of let-7 and miR-200 family members, leading to reversal of the EMT process in pancreatic cancer cells in vitro [44]. Similarly, our results indicated let-7a negatively modulates EMT process in NPC cells. HMGA2 is widely considered as a driver of tumor metastasis and a switching actor of EMT [45-47]. Consistent with the effect of let-7a mimics, knockdown of HMGA2 also suppressed EMT in NPC cells. Based on above result, we thought let-7a negatively modulates EMT process through targeting HMGA2 in NPC cells.

## Conclusions

In summary, our study demonstrated that let-7a was downregulated and inversely associated with the clinical stage, T classification and N classification, and HMGA2 was upregulated and directly associated with the clinical stage and N classification in patients with NPC. Moreover, there was an inverse correlation between let-7a expression and HMGA2 expression in NPC patient. In addition, HMGA2 was negatively regulated at the posttranscriptional level by let-7a via a binding site of HMGA2-3′UTR. In addition, synthetic let-7a mimics suppressed NPC cells migration, invasion and EMT process and knockdown of HMGA2 was consistent with the effects of let-7a in NPC cells. This study suggests that let-7a/HMGA2 may play an important role in tumor metastasis and may be a novel diagnostic marker and potential therapeutic target in NPC.

## Additional files

Additional file 1: Figure S2. The luciferase reporter construct of pLUC. (hRluc: Rellina luciferase, hluc: Firefly luciferase, AMP: Ampicillin). Additional file 2: Figure S1. Quantitative analysis of Western blot. (A) Let-7a mimics inhibited HMGA2 protein expression in NPC cells. (B) HMGA2 protein is reduced by small interfering RNA (HMGA2-siRNA 2) in NPC cells. (C) Let-7a and HMGA2 regulated the expression of MMPs and EMT-associated genes in NPC cells.
