# Supplementary figures and images for: Let-7a inhibits migration, invasion and epithelial-mesenchymal transition by targeting HMGA2 in nasopharyngeal carcinoma
Source: J Transl Med. 2015 Mar 31;13:105. doi: 10.1186/s12967-015-0462-8 (PMC4391148; doi:10.1186/s12967-015-0462-8)

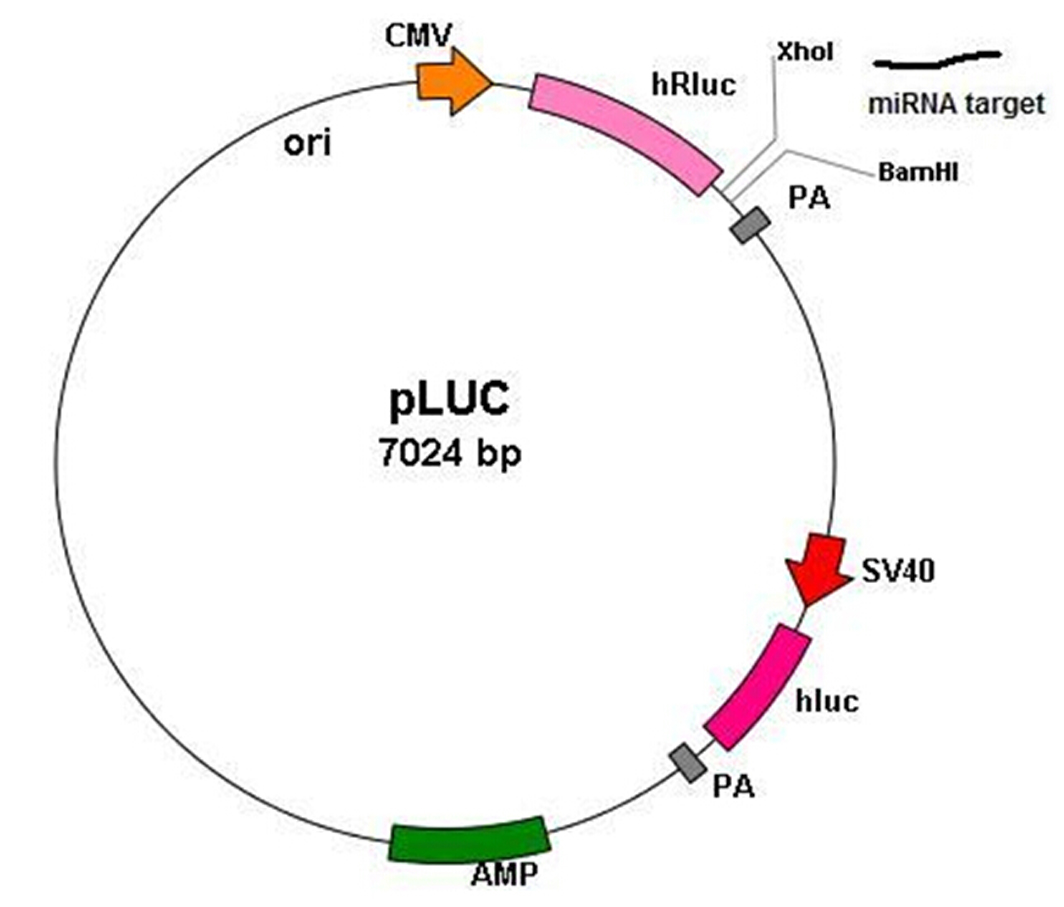

Supplement: Additional file 1: Figure S2. — The luciferase reporter construct of pLUC. (hRluc: Rellina luciferase, hluc: Firefly luciferase, AMP: Ampicillin). [file 12967_2015_462_MOESM1_ESM.jpeg]

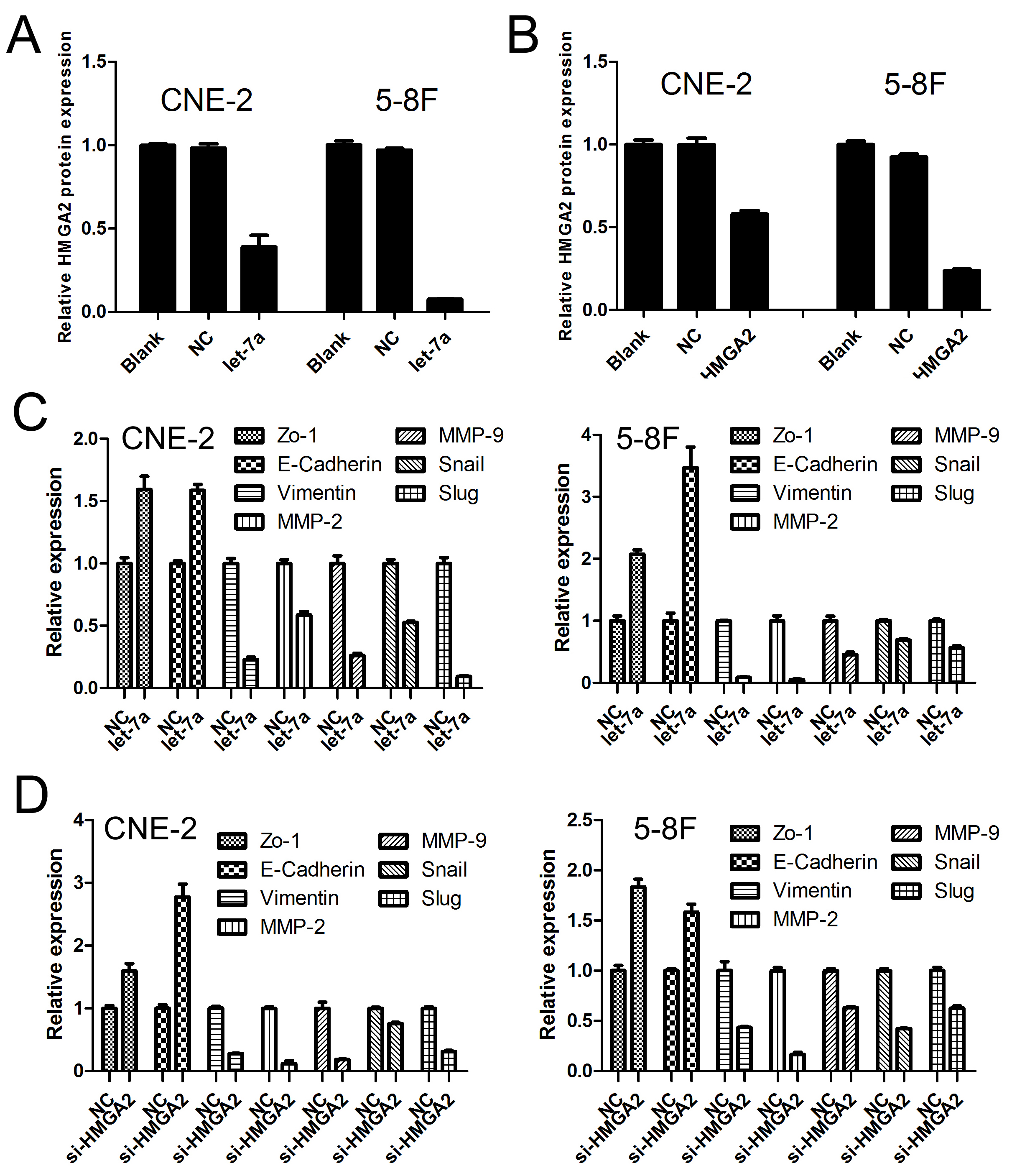

Supplement: Additional file 2: Figure S1. — Quantitative analysis of Western blot. (A) Let-7a mimics inhibited HMGA2 protein expression in NPC cells. (B) HMGA2 protein is reduced by small interfering RNA (HMGA2-siRNA 2) in NPC cells. (C) Let-7a and HMGA2 regulated the expression of MMPs and EMT-associated genes in NPC cells. [file 12967_2015_462_MOESM2_ESM.jpeg]
